# Multi-Physics Coupling Simulation of Surface Stress Waves for Interface Debonding Detection in Underwater Grouting Jacket Connections with PZT Patches

**DOI:** 10.3390/s25103124

**Published:** 2025-05-15

**Authors:** Bin Xu, Qian Liu, Xinhai Zhu, Hanbin Ge

**Affiliations:** 1 College of Civil Engineering, Huaqiao University, Xiamen 361021, China; hqulq@stu.hqu.edu.cn; 2 Key Laboratory for Intelligent Infrastructure and Monitoring of Fujian Province, Huaqiao University, Xiamen 361021, China; 3 Shanghai Municipal Engineering Design Institute (Group) Co., Ltd., Shanghai 200092, China; zhuxinhai@smedi.com; 4 Department of Civil Engineering, Meijo University, Nagoya 468-8502, Japan; gehanbin@meijo-u.ac.jp

**Keywords:** underwater grouting jacket connection (GJC), interface debonding defect, surface wave measurement, wavelet packet energy, piezoelectric lead zirconate titanate (PZT), multi-physical field numerical simulation

## Abstract

Interface debonding between the steel tube and grouting materials in grouting jacket connections (GJCs) of offshore wind turbine supporting structures leads to negative effects on the load-carrying capacity and safety concerns. In this paper, an interface debonding defect detection and localization approach for scale underwater GJC specimens using surface wave measurement is proposed and validated numerically. A multi-physics finite element model (FEM) of underwater GJCs with mimicked interface debonding defects, surrounded by water, and coupled with surface-mounted piezoelectric lead zirconate titanate (PZT) patches is established. Under the excitation of a five-cycle modulated signal, the surface stress wave propagation, including transmission, diffraction, and reflection, within the outer steel tube, grouting material, and inner steel tube is simulated. The influence of mimicked interface debonding defects of varying dimensions on stress wave propagation is systematically analyzed through stress wave field distributions at distinct time intervals. Additionally, the response of surface-mounted PZT sensors in the underwater GJC model under a one-pitch-one-catch (OPOC) configuration is analyzed. Numerical results demonstrate that the wavelet packet energy (WPE) of the surface wave measurement from the PZT sensors corresponding to the traveling path with a mimicked interface debonding defect is larger than that without a defect. To further localize the debonding region, a one pitch and multiple catch (OPMC) configuration is employed, and an abnormal value analysis is conducted on the WPEs of PZT sensor measurements with identical and comparable wave traveling patches. The identified debonding regions correspond to the simulated defects in the models.

## 1. Introduction

The grouting jacket connection (GJC) is an important mechanical transmission component in the jacket structure, responsible for firmly connecting the jacket to the pile foundation. It is not only a key component in supporting the structural load but also a relatively weak link [1,2,3,4]. The GJC comprises a sandwich composite system consisting of a pile guide jacket, a steel pipe pile, and high-performance grouting material between them. The interfacial bonding performance of this system directly affects the service reliability of the entire wind turbine support structure, which is subjected to harsh marine environments and dynamic loads [5,6,7]. Previous studies have documented issues of slip and gap formation at the interface between the steel pipe and the grouting material [8,9]. Schaumann et al. [10] investigated the mechanical properties of grouting connection specimens under fatigue loading, focusing on how interfacial contact conditions between the steel pipe and grouting material affect structural performance. Results indicated that water infiltration at the debonding defects significantly reduces the fatigue performance of the grouting connection. This is primarily because the lubricating effect of water reduces the friction coefficient between the steel pipe and grouting material, leading to stress concentration. Additionally, high-strength grouting material, which acts as the load transfer medium between the steel pipe pile and the upper structure, can compromise the overall load-bearing capacity of the connection segment if debonding occurs. Traditional non-destructive testing (NDT) techniques face challenges in accurately identifying internal defects in GJCs of offshore wind power support structures due to the shielding effect caused by the outer steel tube and the complexities of underwater environments. Therefore, there is an urgent need to develop efficient detection and visualization methods for interface debonding to enhance the detection capabilities for these critical underwater structures.

Traditional NDT methods, such as the hammering method, optical fiber sensing technology, impact echo, electromagnetic imaging, and ultrasonic waves, are widely employed for defect detection in composite structures or composite-reinforced concrete structures [11,12,13,14]. However, inherent limitations in applicability render these methods ineffective for identifying interface defects within steel tubes of steel-concrete composite structures (SCCS) [15]. The rapid development of piezoelectric lead zirconate titanate (PZT) materials in the field of NDT has provided a new method for assessing the interfacial bonding state in SCCS [16,17]. Chen et al. [18] developed a stress wave-based detection method using surface-mounted PZT patches to identify interfacial debonding in SCCS, including concrete-filled steel tube (CFST) components. The basic principle of the detection method was revealed from the investigation of the propagation characteristics of the stress wave in the steel tube. Chen et al. [19] provided a systematic review of advancements in NDT for interface defects in SCCS, emphasizing the potential of stress wave propagation-based methods to identify such defects. To explore the potential of wave measurement in practical engineering applications, Liu et al. [20] conducted debonding defect detection on rectangular CFST specimens that had been stored in the laboratory for over a year. Using surface wave measurements and EMI measurement results from surface-mounted PZT sensors, the artificially mimicked interface defects at unknown positions and the debonding defects in the natural environment are accurately located. Yan et al. [21] used the time reversal technique of stress wave measurement signals to evaluate compactness in CFST components. Wang et al. [22] successfully applied the stress wave measurement technique to detect debonding defects in steel-UHPC composite slabs.

Owing to the unique response characteristics of PZT materials, wave measurement with surface-mounted PZT sensors has emerged as a highly promising method for NDT [23]. Building on this foundation, recent studies have successfully extended the application of wave measurement techniques to the damage assessment of grouted structural components [24,25]. Chen et al. [26] developed a stress wave detection method with surface-mounted PZT transducers, employing time-reversal processing to enhance ultrasonic focusing signals. They established a quantitative correlation between normalized wavelet energy entropy and grouting compactness, significantly improving the signal-to-noise ratio of measurements. However, the feasibility of this approach for interface defect detection in grouting jacket connections (GJCs)—a specialized sandwich composite structure—requires further validation in practical applications. Zhu et al. [27] proposed a PZT sensor-based surface wave method to identify interfacial debonding defects in offshore wind turbine GJCs, achieving regional defect imaging through abnormal signal analysis. Despite these advancements, the effectiveness of this method for the detection of interface defects of GJCs in underwater environments remains to be investigated.

In this paper, aiming at the detection and localization of interface debonding in underwater GJCs of offshore wind turbine support structures, a method based on surface wave measurement is proposed using PZT active actuating and sensing technology. The propagation of stress waves in an underwater GJC model was investigated through multi-physical field coupling finite element analysis with an OPOC configuration. Additionally, the detection mechanism of interface debonding defects based on surface wave measurements is explored. The influence of interface debonding defects on surface wave propagation is illustrated through stress wave fields at various time instants. The difference in surface wave measurements from surface-mounted PZT sensors, corresponding to wave traveling paths with and without debonding defects, is analyzed. Based on the abnormal value analysis of the PZT sensor measurements, the surface wave paths affected by interface debonding defects are identified, enabling the evaluation of the debonding region. The results indicate that the assessed debonding region aligns accurately with the defect locations in the numerical models.

## 2. Multi-Physics Coupling Analysis of Surface Wave Propagation

### 2.1. Multi-Physics Finite Element Modeling for PZT–GJC–Water Coupling System

PZT materials offer a novel non-destructive evaluation approach for assessing the interfacial bond condition in underwater GJCs, leveraging their cost-effectiveness, rapid response, and dual actuation–sensing capabilities. When an alternating voltage is applied to a PZT actuator mounted on the surface of the outer steel pipe, the converse piezoelectric effect of the material induces longitudinal vibrations along the long axis of the PZT patch, generating stress waves within the wall of the steel tube. These stress waves propagate along the steel tube and reach the PZT sensor, where the vibration is converted into an electrical signal by the positive piezoelectric effect of the material. When the interfacial bonding between the actuator and sensor is intact, the stress waves propagate along the steel tube in the form of Rayleigh waves, simultaneously inducing vibrations in the concrete core. This ultimately results in the penetration of the energy of the surface waves from the steel pipe into the concrete. When a debonding defect exists at the interface, the Rayleigh waves in the steel tube transform into Lamb waves at the onset of debonding and propagate along the steel tube, reverting back to Rayleigh waves at the end of the debonding defect. The interfacial debonding defect causes the steel tube to lose its constraint on the concrete core at the deboned location, significantly reducing the stress wave energy transmitted from the steel tube to the concrete. As a result, the amplitude of the voltage signals measured by the PZT sensors along the propagation path increases [18].

In this paper, a three-dimensional electromechanical coupling finite element model of the surrounding water, the PZT patches, and the GJC section is established using COMSOL 6.0 software. The solid mechanical field in the structural mechanics module, the electrostatic field in the electric field, and the pressure acoustics field are selected to carry out transient analysis. In the numerical simulation, the dynamic equation and electrical state equation for the PZT patch are represented by Equations (1) and (2), respectively [18].

(1) $$\left[M\right]\left\{\ddot{u}\right\}+\left[K\right]\left\{u\right\}=V\left\{P\right\}+\left\{F\right\}$$

(2) $${\left\{P\right\}}^{T}\left\{u\right\}+CV=Q$$

Equation (3) is the governing equation for the coupling system considering the piezoelectric effect.

(3) $$\left[\begin{array}{cc} \left[M\right] & \left[0\right]\\ \left[0\right] & \left[0\right] \end{array}\right]\left\{\begin{array}{c} \ddot{\mu }\\ \ddot{V} \end{array}\right\}+\left[\begin{array}{cc} \left[C\right] & \left[0\right]\\ \left[0\right] & \left[0\right] \end{array}\right]\left\{\begin{array}{c} \dot{\mu }\\ \dot{V} \end{array}\right\}+\left[\begin{array}{cc} \left[K\right] & \left[K^{Z}\right]\\ {\left[K^{Z}\right]}^{T} & \left[K^{d}\right] \end{array}\right]\left\{\begin{array}{c} \mu \\ V \end{array}\right\}=\left\{\begin{array}{c} \left\{F\right\}\\ \left\{Q\right\} \end{array}\right\}$$

When the PZT patch is used as an actuator,

(4) $$\left\{F\right\}=\left\{0\right\},\left\{Q\right\}=\left\{Q(t)\right\}$$

Accordingly,

(5) $${\left[K^{Z}\right]}^{T}\left\{u\right\}+\left[K^{d}\right]\left\{V\right\}=\left\{Q(t)\right\}$$

(6) $$\left[M\right]\left\{\ddot{u}\right\}+\left[C\right]\left\{\dot{u}\right\}+\left(\left[K\right]\left\{u\right\}+\left[K^{Z}\right]\left\{V\right\}\right)=0$$

When the PZT patch is used as a sensor,

(7) $$\left\{F\right\}=\left\{F(t)\right\},\left\{Q\right\}=\left\{0\right\}$$

Accordingly,

(8) $$\left[M\right]\left\{\ddot{u}\right\}+\left[C\right]\left\{\dot{u}\right\}+\left\{\left[K\right]-\frac{{\left[K^{Z}\right]\left[K^{Z}\right]}^{T}}{\left[K^{d}\right]}\right\}\left\{u\right\}=\left\{F(t)\right\}$$

(9) $$\left\{V\right\}=-\frac{{\left[K^{Z}\right]}^{T}\left\{u\right\}}{\left[K^{d}\right]}$$

Due to space limitations, the specific meanings of the symbols in the equation are consistent with those in the study by Chen et al. [19].

The governing equation for the pressure acoustics field is the time-domain wave equation, as shown in Equation (10),

(10) $$\frac{1}{\rho c^{2}}\frac{\partial^{2}p_{t}}{\partial t^{2}}+\nabla \cdot \left(-\frac{\nabla p_{t}}{\rho }\right)=0$$

Here, ρ is the density of the medium, $p_{t}$ is the total acoustic pressure, and *c* is the speed of sound.

**Figure 1:**
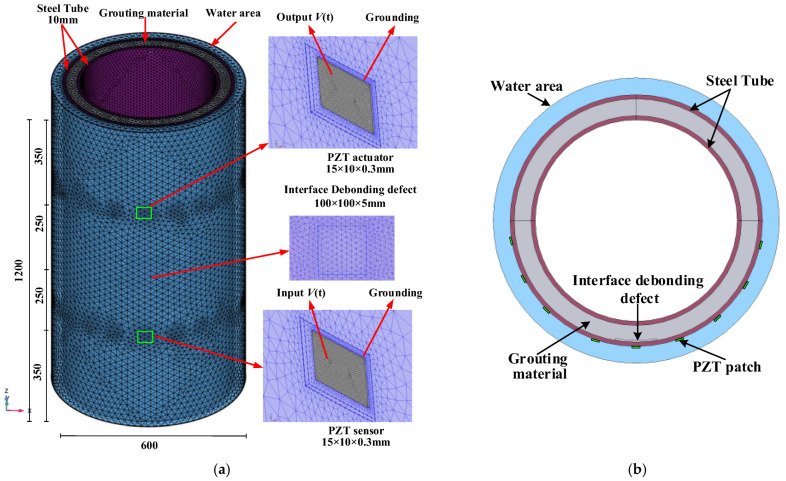
Multi-physics coupled finite element model: (**a**) underwater GJC model; (**b**) cross-sectional diagram.

As depicted in Figure 1, the underwater GJC model has a height of 1200 mm, and the thickness of the two steel tubes is 10 mm. The inner and outer steel tubes of the underwater GJC model have diameters of 500 mm and 600 mm, respectively. The steel tube material is Q235, and a high-strength, non-shrink grouting material with a C60 strength grade is poured between the inner and outer steel tubes. The surrounding water, with a radial thickness of 40 mm, was established outside the GJC to simulate the underwater environment. A free tetrahedral meshing approach was used to mesh the coupling model, with refined finite element meshing around the PZT patch to improve calculation accuracy. The complete coupling model consists of 1,755,716 elements, as illustrated in Figure 1. Both the PZT actuator and sensor used in this study share the same dimensions of 15 mm × 10 mm × 0.3 mm and are polarized along their thickness direction. The detailed mechanical property parameters (elastic constants) and piezoelectric property parameters (dielectric constants and piezoelectric stress constants) of the employed PZT sensors are referenced from previous studies [18], and the specific material parameters are shown in Table 1.

Given that the multi-physics coupling model is a portion of the GJC structure, low-reflection boundaries were defined at the upper and lower surfaces of the model to reduce reflections from the surface boundaries. Additionally, considering that the model is surrounded by an infinite water domain, plane wave radiation boundaries were applied to the upper and lower surfaces and the outer side of the water area to reduce reflections when stress waves reach the outer boundary of the water area. Based on the propagation mechanism and attenuation characteristics of stress waves in the GJC component, a 5-cycle sinusoidal windowed signal with an amplitude of 10 V and a frequency of 20 kHz was applied to the upper surface of the PZT actuator. At the interface where the PZT patch is coupled with the outer steel tube, the voltage was set to 0 V (ground).

The low-reflective boundary conditions for the GJC component coupled with the PZT actuator and sensor satisfy the following equations.

(11) $$\sigma \cdot n=-d_{im}\frac{\partial u}{\partial t}$$

(12) $$d_{im}=d_{im}\left(K,c_{s},c_{p}\right)$$

Here, **σ** is the stress tensor, **n** is the normal vector to the boundary, and *d_im_* represents a function, where *K* denotes the density, *c_s_* is the shear wave velocity, and *c_p_* is the longitudinal wave velocity.

The plane wave radiation boundary conditions, which are employed to minimize the reflection of stress waves at the exterior boundaries of the water, are governed by the following equations.

(13) $$-n\cdot \left(-\frac{\nabla p_{t}}{\rho }\right)+\frac{1}{\rho }\left(\frac{1}{c}\frac{\partial p}{\partial t}\right)=0$$

Here, **n** is the normal vector to the boundary, ρ is the density of the medium, and $p_{t}$ is the total acoustic pressure.

### 2.2. Arrangement of Interface Debonding Defects

Due to the invisibility of the grouting process, complex offshore construction conditions, vertical gravitational loads from the superstructure and equipment, dynamic loads from waves, and other adverse factors, interface debonding and even slippage can easily occur between the grouting material and the steel tube, posing safety hazards. To verify the effectiveness of the surface wave measurement method, gaps of varying sizes were introduced between the grouting material and the steel tube in the finite element model using Boolean operations to simulate interfacial debonding defects. The radial thickness of each defect was set to 5 mm.

The center of the defect is 600 mm from the bottom surface of the model. To investigate the influence of different bonding conditions between the steel tube and the grouting material on surface wave propagation and the measurement signals of PZT sensors, various interface debonding defect dimensions were set in the underwater GJC model, as shown in Table 2. The dimensions of the defects primarily depend on two variables: vertical height and circumferential length. Therefore, the dimensions of the mimicked interface debonding defects, D1, D2, and D3, are 50 mm × 100 mm × 5 m, 100 mm × 100 mm × 5 m, and 15 mm × 100 mm × 5 m, respectively, to consider variations in the vertical height of the debonding defects. The dimensions of interface debonding defects, D4, D2, and D5, are 100 mm × 50 mm × 5 m, 100 mm × 100 mm × 5 m, and 100 mm × 150 mm × 5 m, respectively, to consider variations in the circumferential length of the interface debonding. All the mimicked interface defects have an identical thickness of 5 mm.

### 2.3. Time-Domain Analysis of Stress Wave Fields and Propagation

In the finite element analysis, the one pitch and one catch (OPOC) and one pitch and multiple catches (OPMC) surface wave measurement configurations are employed respectively for the purpose of identifying and determining the region of the interface debonding defects.

For surface wave measurements using the OPOC measurement configuration, PZT patches are arranged on the outer surface of the outer steel tube at various heights of the GJC model, positioned at a vertical distance of 250 mm from the center of the interface debonding defect. The PZT actuator (designated as P) is installed at 350 mm from the bottom of the GJC model, and the PZT sensor (designated as S) is installed at 850 mm from the bottom. To facilitate a comparative analysis of surface wave measurements, a GJC model without an interface debonding defect has been established. The output signal from the PZT sensor in this model is labeled as “health”.

**Figure 2:**
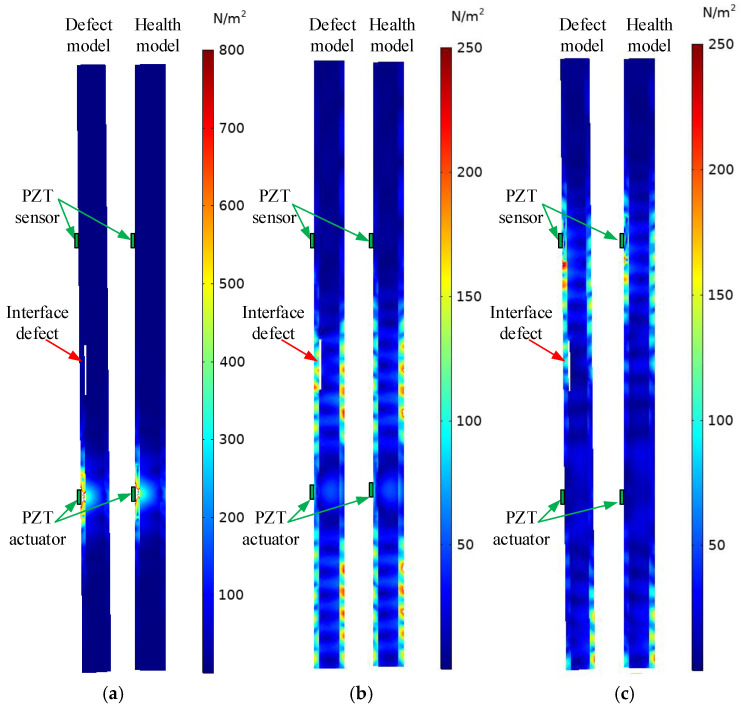
Comparison of stress waves in the vertical section of underwater GJCs model with and without an interface debonding defect at different time instants: (**a**) t_1_ = 1.0 × 10^−4^; (**b**) t_2_ = 2.64 × 10^−4^; (**c**) t_3_ = 3.8 × 10^−4^.

**Figure 3:**
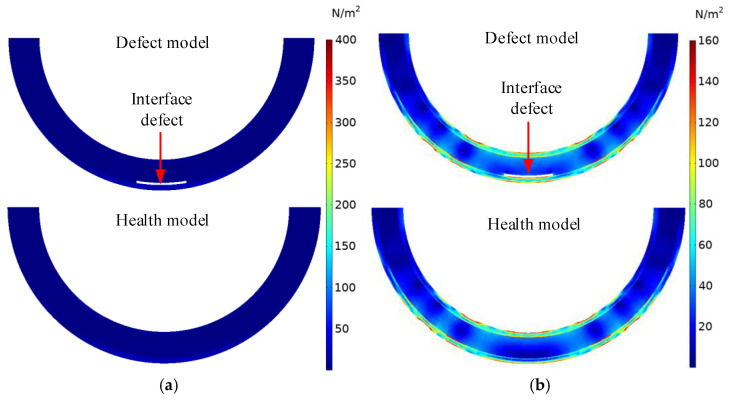
Comparison of stress waves in the horizontal section of underwater GJCs model with and without an interface debonding defect at different time instants: (**a**) t_1_ = 1.0 × 10^−4^; (**b**) t_2_ = 2.64 × 10^−4^.

**Figure 4:**
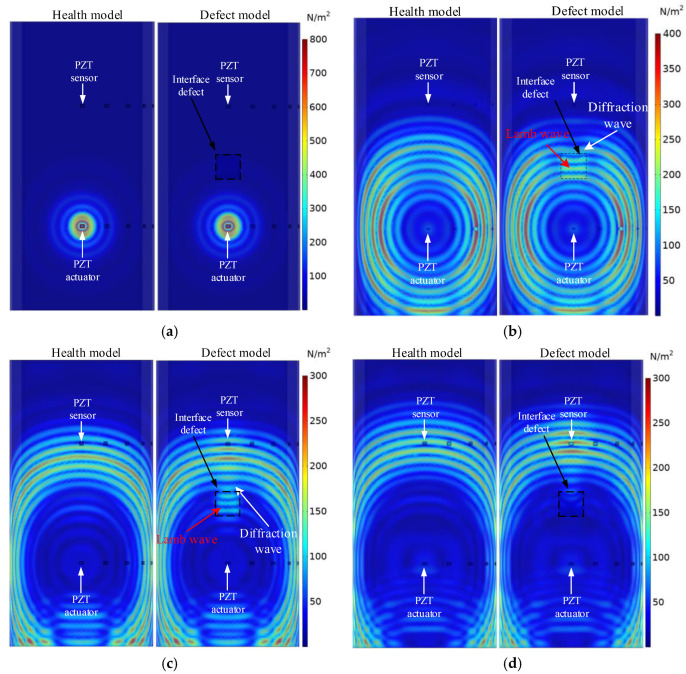
Comparison of stress waves in underwater GJCs model with and without an interface debonding defect at different time instants: (**a**) t_1_ = 1.0 × 10^−4^; (**b**) t_2_ = 2.88 × 10^−4^; (**c**) t_3_ = 3.8 × 10^−4^; (**d**) t_4_ = 4.2 × 10^−4^.

The underwater GJC component consists of the outer steel tube, grouting material, inner steel tube, and the surrounding water environment. A finite element analysis was conducted to investigate the characteristics of stress waves excited by the surface-mounted PZT patch and the propagation process of stress waves within the underwater GJC model. The differences in the stress wave inside the underwater GJC model with and without interface debonding defects were observed at different time instants. To comprehensively observe the propagation process of the stress wave in the 3D underwater GJC model, a five-cycle sinusoidal windowed excitation signal (10 V amplitude, 20 kHz frequency) was applied. The time history of stress wave propagation was analyzed across three perspectives: vertical section, horizontal section, and overall model, with specific results shown in Figure 2, Figure 3 and Figure 4.

The propagation process of stress waves in the vertical section of the underwater GJC model is shown in Figure 2, both with and without interface debonding defects. At t_1_ = 1.0 × 10^−4^ s, the stress wave has not yet reached the boundary of the interface debonding defect. The comparison of the wavefields in the underwater GJC models with and without the interface debonding defect is shown in Figure 2a. Stress waves propagate as Rayleigh and body waves, with body waves traveling through the grouting material and generating reflected and transmitted waves at the grouting material–inner steel tube interface. At this stage, no discernible differences are observed between the wavefields of the defective and intact models. At t_2_ = 2.64 × 10^−4^ s, significant differences in the wavefield are observed in the underwater GJC model with and without interface debonding, as shown in Figure 2b.

In the underwater GJC model with an interface debonding defect, Rayleigh waves propagate along two distinct paths. One part of the wave continues to propagate forward as diffraction waves around the edge of the debonding defect, while the other part propagates as typical Lamb waves in the outer steel tube at the location of the defect. The wave energy in the outer steel tube is notably higher in the defective model compared to the healthy model, indicating that the interface debonding defect weakens the efficiency of energy transfer from the outer steel tube to the grouting material. At t_3_ = 3.8 × 10^−4^, when the surface wave propagates across the interface debonding, it initially propagates as a Lamb wave along the steel tube from the beginning of the debonding to the top end of the defect. Subsequently, the surface wave gradually transitions to a new Rayleigh wave that continues to propagate in the well-bonded region of the outer steel tube. Analyzing the wavefields of stress waves at different times helps to understand the propagation process of stress waves in underwater GJC models with and without interface debonding defects. Results demonstrate that the existence of the interface debonding defect causes surface waves to propagate in the form of Lamb waves in the outer steel tube, attenuating energy transmission to the grouting material. This result implies the feasibility of the interface debonding defect detection method based on surface wave measurements for underwater GJCs.

The comparison of the stress wave propagation process in the horizontal section passing through the center of the interface debonding defect in the underwater GJC models is shown in Figure 3, both with and without the defect at different time instants. At t_1_ = 1.0 × 10^−4^, the stress wave has not yet arrived at the center of the defect; no significant difference between the waveforms in both models can be detected. At t_2_ = 2.64 × 10^−4^, as the stress wave traverses the debonding interface, distinct waveforms emerge in both models, with discernible disparities in their wavefield. Consistent with the wave field results in Figure 2, the Lamb wave propagating in the middle of the interface debonding defect can be observed, and the peak value of the stress wave in the outer steel tube at the defect location is markedly higher than in the corresponding region of the healthy model.

Figure 4 compares stress wave propagation in the outer steel tube of the underwater GJCs models with and without interface debonding defects at different time instants. As depicted in Figure 4a, prior to the stress wave reaching the debonding interface, no significant differences in propagation behavior are observed between the two models, both exhibiting similar waveforms and wavefronts. As shown in Figure 4b,c, for the underwater GJC model with interface debonding defects, the stress wave in the outer steel tube at the defect location is in the form of a Lamb wave, while the wave passing through the edge of the interface defect continues to propagate forward as a diffraction wave. Compared to the healthy model, the stress wave strength in the outer steel tube at the top of the defect is significantly greater, indicating that the existence of the interface defect weakens the energy transfer from the outer steel tube to the grouting material. At t_4_ = 4.2 × 10^−4^ s, the defective model exhibits substantially greater stress wave amplitude than its healthy counterpart. Furthermore, wavefield comparisons reveal smaller amplitude variations in the healthy model, underscoring the pronounced influence of interface debonding on stress wave propagation. These observations validate the feasibility of employing the OPMC configuration for interface debonding detection in underwater GJCs.

Through three-dimensional finite element simulations, the propagation of surface waves in underwater GJCs was analyzed, and the influence of interface debonding defects on the stress wave time history and t wavefield energy distribution was investigated. The results indicate that interface debonding alters the propagation path, wave type, and energy distribution of surface stress waves. This provides a theoretical foundation for detecting interface debonding defects in underwater GJCs based on surface wave measurements.

## 3. Simulation Response of PZT Sensor Measurements with OPOC Configuration

### 3.1. Effect of Vertical Heights of Debonding Defects

**Figure 5:**
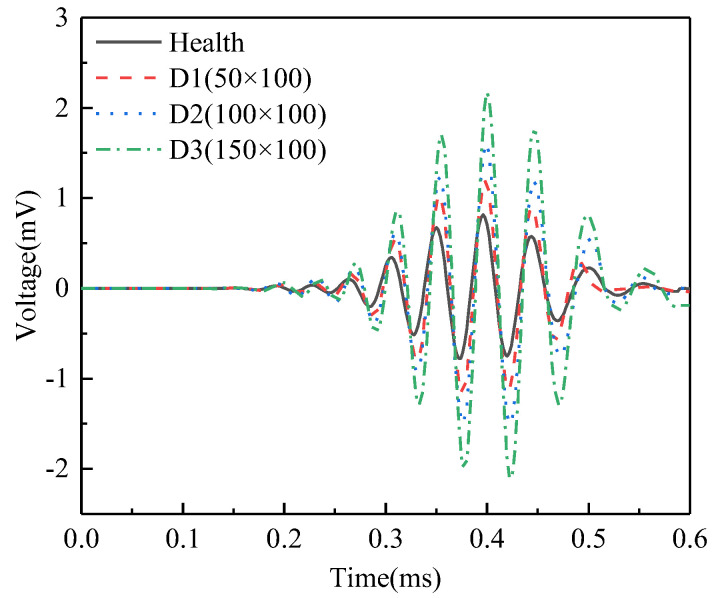
Comparison of the time-domain signals of the PZT sensor measurements corresponding to GJC models with debonding defects of different vertical heights.

Figure 5 compares the voltage signals output by the PZT sensor in the underwater GJC model with interface debonding defects of different vertical heights. It can be observed that there are significant differences in the signal amplitudes output by the PZT sensor between underwater GJC models with different interface bonding conditions. Specifically, the signal amplitude in the healthy GJC model is lower than that in the GJC with interface debonding defects. Furthermore, Furthermore, as the vertical height of the debonding defects increases, the PZT sensor output amplitude rises markedly, demonstrating a direct correlation between defect severity and signal magnitude.

**Figure 6:**
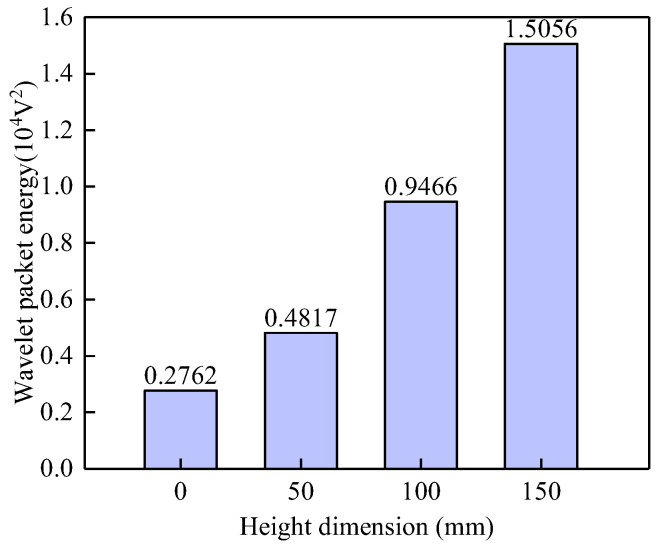
Comparison of the WPE values of the PZT sensor measurements corresponding to GJC models with debonding defects of different vertical heights.

Through wavelet analysis of the output voltage signals from PZT sensors, this study investigates the influence of the vertical height of interface debonding defects on the wavelet packet energy (WPE) values of the signals, aiming to quantify damage severity. Based on the WPE calculation method proposed in Reference [15], Figure 6 presents the comparative results of WPE values of the signals from the surface-mounted PZT sensors in the underwater GJC models under different vertical heights of debonding defects. It can be clearly observed from the figure that there is a significant difference in WPE values between the healthy model and the models with interfacial debonding defects, with the healthy condition exhibiting the smallest WPE value. This finding aligns with the previously analyzed stress wave field results, which indicated that interfacial debonding defects substantially reduce energy transmission efficiency in the steel tube, thereby increasing the WPE values of signals from surface-mounted PZT sensors. Further analysis reveals that the WPE values are highly sensitive to changes in the vertical height of the debonding defects. As the vertical height of the debonding defect increases from D1 (50 mm) to D3 (150 mm), the corresponding WPE values exhibit an approximately linear growth trend, with the highest WPE value observed under the D3 defect condition. These findings validate the effectiveness of the proposed surface wave-based method for identifying interfacial debonding defects in underwater GJCs, confirming a strong positive correlation between wavelet packet energy and the vertical height of the debonding defect.

### 3.2. Effect of Circumferential Dimensions of Debonding Defects

**Figure 7:**
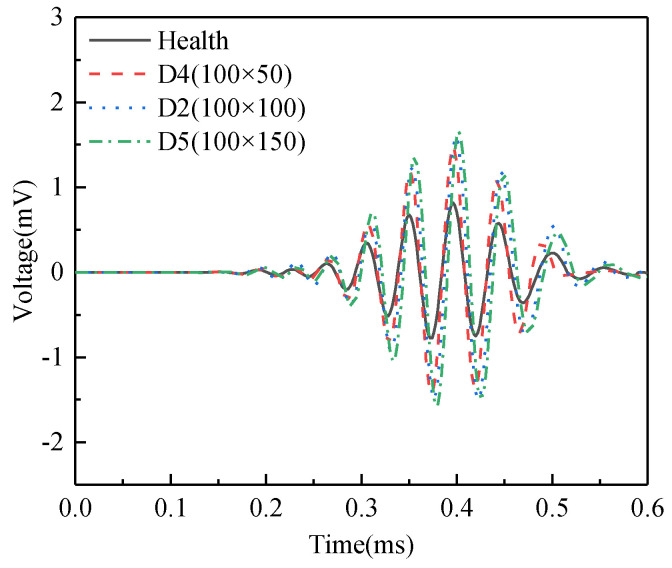
Comparison of the time-domain signals of the PZT sensor of underwater GJC models with interface debonding defects of different circumferential dimensions.

Figure 7 illustrates the comparison of the voltage signals from the PZT sensor in underwater GJC models with interface debonding defects of different circumferential dimensions. Similar to the results shown in Figure 5, the amplitudes of the response of surface-mounted PZT sensors in the underwater GJC models with debonding defects are significantly larger than those in the GJC model without defects. These results confirm that surface wave measurements using the OPOC configuration enable accurate identification of interface debonding defects. However, as the circumferential dimension of the defect increases, amplitude differences between models with varying defect sizes diminish, suggesting limited sensitivity to circumferential defect scaling under the tested conditions.

**Figure 8:**
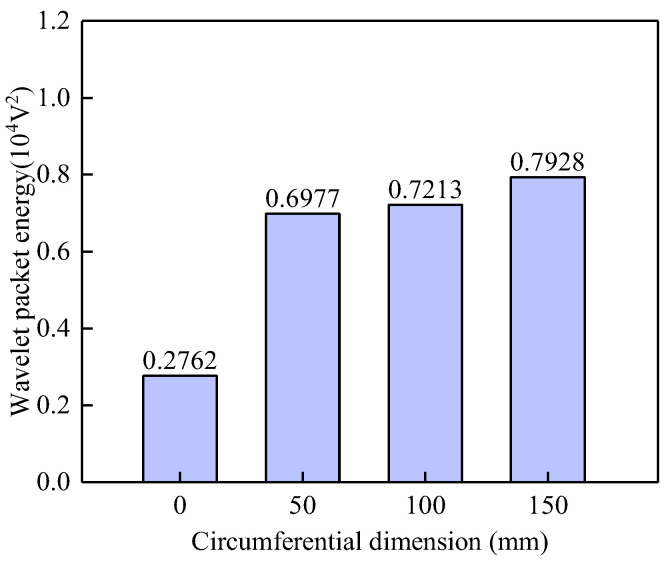
Comparison of the WPE values of the PZT sensor of underwater GJC models with debonding defects of different circumferential dimensions.

Figure 8 compares the WPE values of signals from surface-mounted PZT sensors in GJC models with varying circumferential dimensions of debonding defects. The figure clearly shows that the WPE values of the PZT sensor output signals are highly sensitive to the existence of interface debonding defects, even for defects with a height of 50 mm and a circumferential size of 100 mm. However, WPE values show limited sensitivity to changes in circumferential defect size. As the circumferential dimension of the debonding defect increases from 50 mm to 150 mm, the corresponding WPE values exhibit a gentle growth trend with minimal differences. These findings further validate the effectiveness of the proposed method for identifying interfacial debonding defects in underwater GJCs.

## 4. Multi-Physics Coupling Analysis on Measurement of Underwater GJCs with Interface Debonding for Detection and Localization

### 4.1. OPMC Configuration for Surface Wave Measurement

**Figure 9:**
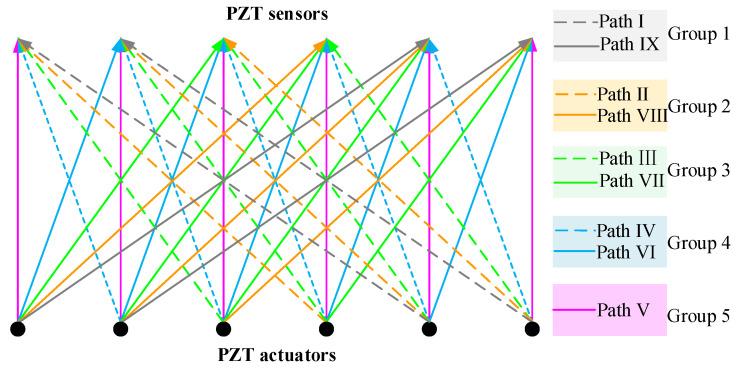
OPMC measurement configuration and paths definition.

In practice, the actual region of interface debonding defects in GJC specimens is typically unknown before testing. It is desirable to develop an approach for identifying and localizing debonding regions or distributions in GJC specimens using surface wave propagation measurements. Therefore, an OPMC surface wave measurement configuration is proposed to identify the regions of interface debonding defects in GJCs. In the OPMC configuration, one surface-mounted PZT actuator is excited as an actuator, and nine surface-mounted PZT sensors are employed to measure the surface wave. The surface wave traveling paths with identical stress wave traveling distances and directions are grouped and color-coded, as illustrated in Figure 9. The nine paths originating from the actuator are categorized into five distinct groups. Anomaly detection analysis is then applied to each group to isolate paths exhibiting abnormal wave propagation behavior, enabling precise defect localization.

**Figure 10:**
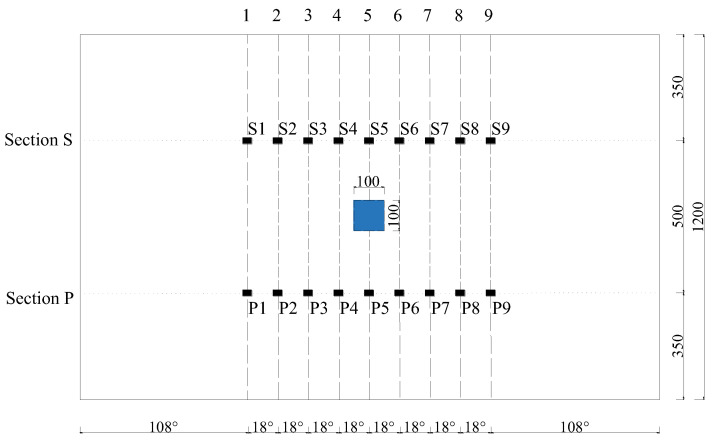
Position unfolded diagram of defect and PZT patches in the finite element model.

For surface wave measurements using the OPMC configuration, PZT actuators and sensors are mounted at 100 mm intervals along the circumferential direction of the outer surface of the model. The actuators and sensors are arranged in sections P and S, respectively. The layout of the positions of the interface debonding defect and PZT patches in the finite element model is shown in Figure 9. The measurement method of a pitch and nine catches is used to obtain the output signal of the PZT sensor for each group of traveling paths. Wavelet packet energy calculations are performed on the measured signals of each traveling path with identical measurement distance and direction, followed by abnormal value analysis. The PZT actuators are numbered P1–P9, the PZT sensors are numbered S1–S9, and the measurement paths are defined in Figure 10.

### 4.2. Simulation Results of OPMC Measurement Configuration

To validate the efficacy of the defect range identification method based on the OPMC configuration for surface stress wave measurement, a numerical study was conducted on an underwater GJC featuring a 100 mm × 100 mm × 5 mm interface debonding defect. The arrangement of the defects and PZT patches is shown in Figure 10. As numerically validated earlier, interface debonding alters wave propagation, enabling elevated wavelet packet energy (WPE) values within specific traveling path groups to serve as indicators of potential defects. Therefore, the intersection region covered by the identified abnormal surface wave traveling paths can be treated as an estimation of interface debonding defects in the GJC specimens.

#### 4.2.1. Judgment of Abnormal Surface Wave Traveling Paths

The abnormal values are identified based on a statistics-based abnormal data analysis approach. Unlike the method described in the “Technical specification for inspection of concrete defects by ultrasonic method” (CECS 21-2000), which classifies smaller values as abnormal, this study defines values exceeding a predetermined threshold as abnormal, with corresponding surface wave traveling paths indicating potential interface debonding defects [28].

Using one pitch and nine catch configurations, surface wave propagation paths with identical stress wave traveling distances and directions are grouped. Subsequently, the judgment value of the WPE value for each group is determined using the abnormal value analysis method. By comparing and analyzing the time-domain signals of each path within each group, the abnormal paths are preliminarily identified. Paths with WPE values surpassing the judgment value are identified as abnormal, signifying debonding defects along those trajectories.

In each group, the WPE values derived from surface wave measurements were sorted in ascending order, represented as *X*_1_ ≤ *X*_2_ ≤ … ≤ *X_n_* ≤ *X_n_*_+1_. Data points significantly larger than adjacent ones in line are judged as suspicious data points. The smallest candidate suspicious data point (denoted as *X_n_*) and its preceding data points are subjected to statistical analysis. The arithmetic mean (*m_x_*) and standard deviation (*s_x_*) are calculated using Equations (14) and (15), respectively. These parameters are subsequently substituted into Equation (16) to derive the judgment value *X*_0_, which serves as the discriminant boundary for abnormal data classification.

(14) $$m_{x}=\Sigma X_{i}/n$$

(15) $$s_{x}=\sqrt{\left(\Sigma X_{i}^{2}-n\cdot m_{x}^{2})/(n-1\right)}$$

(16) $$X_{0}=m_{x}+\lambda_{1}\cdot s_{x}$$

Here, *λ*_1_ is derived from the specification [28], *X_i_* represents the *i*-th measurement value, and *n* denotes the total number of data points included in the statistical analysis.

The judgment value *X*_0_ is compared with the minimum value *X_n_* of the suspicious data. If *X*_0_ ≤ *X_n_*, *X_n_* and all subsequent data points (*X_n_*, *X_n_*_+1_,…) are classified as abnormal values and removed. The remaining dataset (*X*_1_~*X_n_*_−1_) is then iteratively reanalyzed using Equations (14)–(16) until no further abnormal values are detected. Conversely, if *X*_0_ > *X_n_*, the next ordered value *X_n_*_+1_ should be included in the calculation and re-evaluation process.

**Figure 11:**
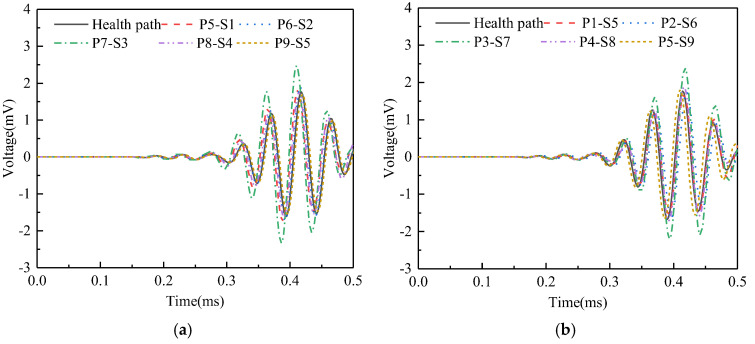
Time domain signal of PZT sensors output signal of Group 1: (**a**) path I; (**b**) path IX.

**Figure 12:**
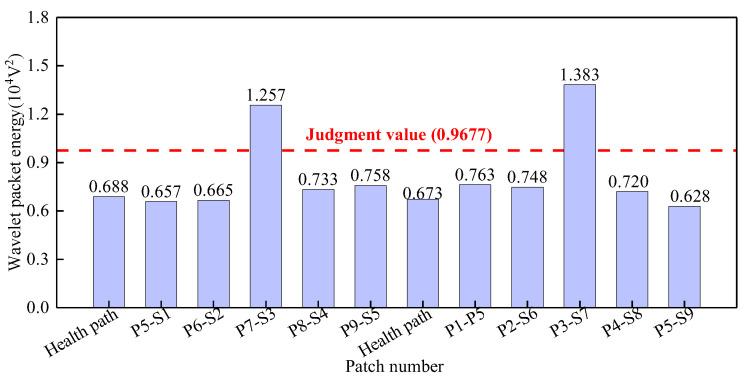
The wavelet packet energy of PZT sensors output signal of Group 1.

As illustrated in Figure 9, surface wave measurement paths I and IX are categorized into Group 1. The corresponding PZT sensor responses, shown in Figure 11, reveal that the time-domain signal peaks of the measurement paths P7-S3 and P3-S7 are significantly larger than those of the remaining paths. The wavelet packet energy calculation was performed on the output signal of the PZT sensor, and the abnormal value judgment was made. The results are shown in Figure 12.

As shown in Figure 12, the wavelet packet energy judgment value for Group 1 is 0.9677 × 10^−4^ V^2^. The WPE values of the signals on path P7-S3 and path P3-S7 are obviously larger than this judgment value and can be judged as abnormal values. Therefore, it is determined that path P7-S3 and path P3-S7 are abnormal surface wave traveling paths with interface debonding defects.

**Figure 13:**
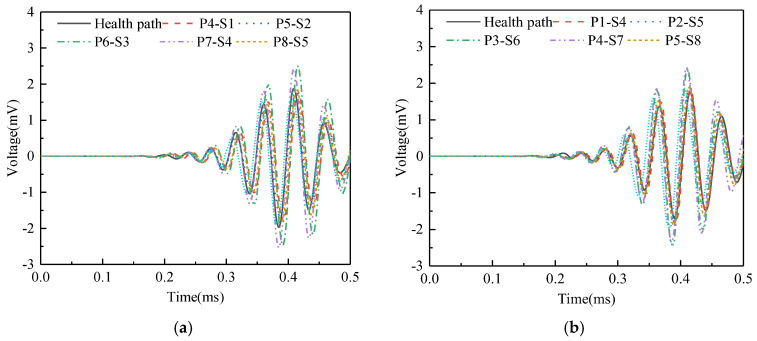
Time domain signal of PZT sensors output signal of Group 2: (**a**) path II; (**b**) path VIII.

**Figure 14:**
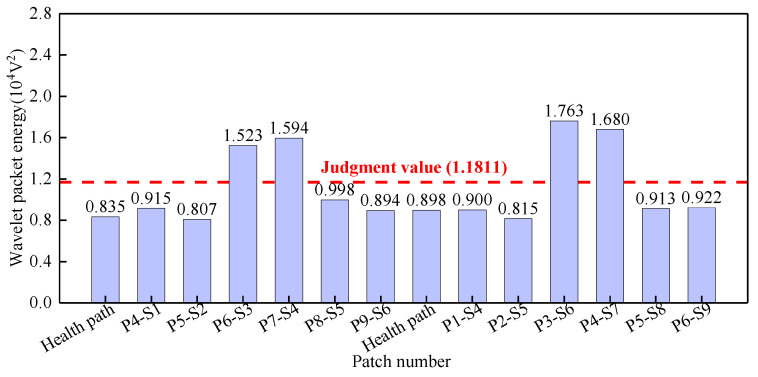
The wavelet packet energy of PZT sensors output signal of Group 2.

Surface wave measurement paths II and VIII are grouped into Group 2. The corresponding PZT sensor responses, shown in Figure 13, reveal that time-domain signal peaks for paths P6-S3, P7-S4, P3-S6, and P4-S7 significantly exceed those of the remaining paths. WPE calculations were performed on the output signal of the PZT sensors, and the abnormal value judgment was made, with results detailed in Figure 14. The WPE judgment value for Group 2 is 1.1811 × 10^−4^ V^2^. The WPE values of the PZT sensor output signals on paths P6-S3, P7-S4, P3-S6, and P4-S7 are obviously larger than the judgment value and can be judged as abnormal values. The identification of these four abnormal surface wave traveling paths confirms the presence of interface debonding defects.

**Figure 15:**
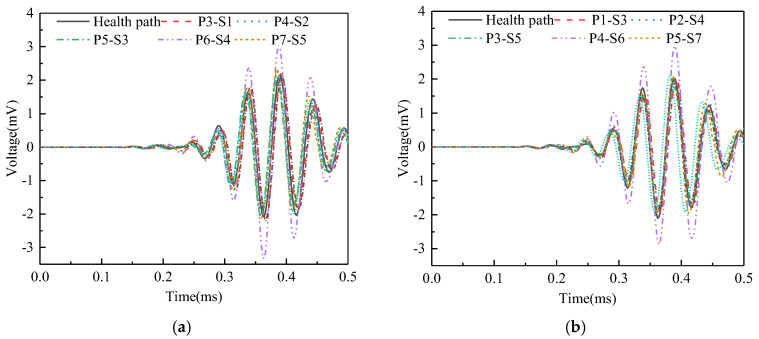
Time domain signal of PZT sensors output signal of Group 3: (**a**) path III; (**b**) path VII.

**Figure 16:**
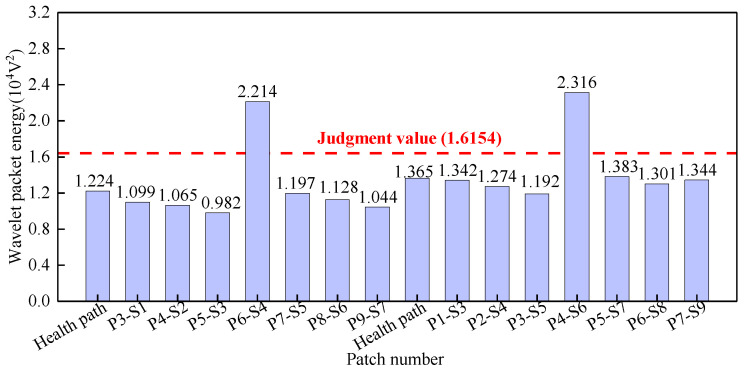
The wavelet packet energy of PZT sensors output signal of Group 3.

Surface wave measurement paths III and VII are categorized into Group 3. The corresponding PZT sensor responses, illustrated in Figure 15, demonstrate that time-domain signal peaks for paths P6-S4 and P4-S6 significantly exceed those of the remaining paths. Abnormal value analysis was performed on the WPE of the output signals from the PZT sensors, and the results are shown in Figure 16. The WPE judgment value for Group 3 is 1.6154 × 10^−4^ V^2^. The WPE values of the PZT sensor output signals on paths P6-S4 and P4-S6 are obviously larger than the judgment value and can be judged as abnormal values. Therefore, paths P6-S4 and P4-S6 are considered to be abnormal surface wave traveling paths with interface debonding defects.

**Figure 17:**
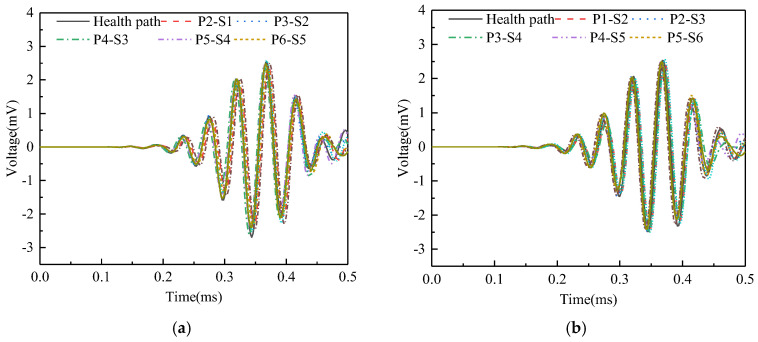
Time domain signal of PZT sensors output signal of Group 4: (**a**) path IV; (**b**) path VI.

**Figure 18:**
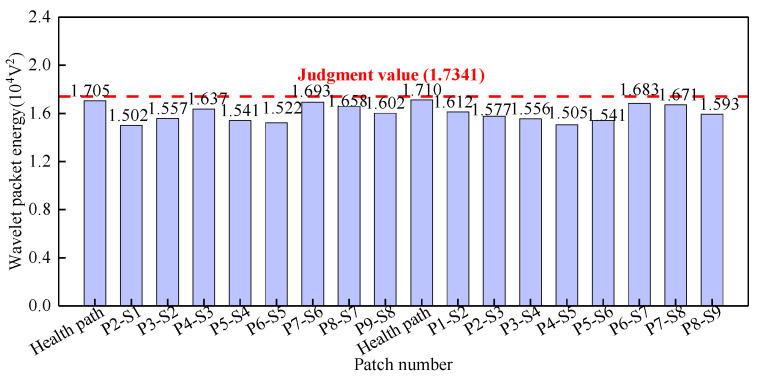
The wavelet packet energy of PZT sensors output signal of Group 5.

Surface wave measurement paths IV and VI are grouped into Group 4. The corresponding PZT sensor responses, shown in Figure 17, reveal no significant variation in time-domain signal peaks across all propagation paths. The abnormal value analysis was performed on the WPE of the output signal from the PZT sensors, and the results are shown in Figure 18. The WPE judgment value for Group 4 is 1.7341 × 10^−4^ V^2^. The WPE values of all paths are less than the judgment value, indicating the absence of interface debonding defects in the surface wave traveling paths IV and VI.

**Figure 19:**
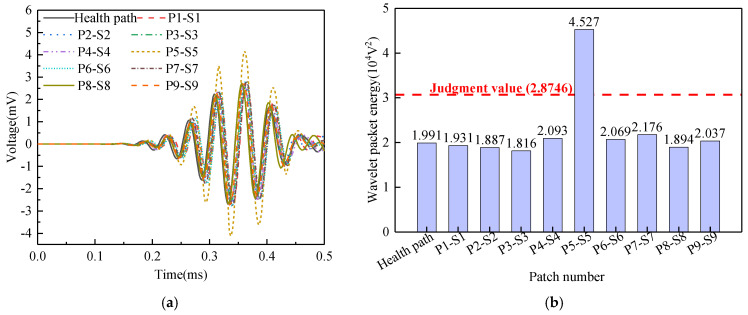
PZT sensors output signal of Group 5: (**a**) time-domain signal; (**b**) wavelet packet energy.

Surface wave measurement path V are grouped into Group 5. The corresponding PZT sensor responses, shown in Figure 19a, reveal that the time-domain signal peak for path P5-S5 significantly exceeds those of the remaining paths. Abnormal value analysis was performed on the WPE, and the results are shown in Figure 19b. The WPE judgment value for Group 5 is 2.8746 × 10^−4^ V^2^. The WPE value for path P5-S5 (4.527 × 10^−4^ V^2^) markedly exceeds this judgment value, classifying it as abnormal. Therefore, an interface debonding defect can be detected in the surface wave traveling path P5-S5.

#### 4.2.2. Debonding Region Localization

**Figure 20:**
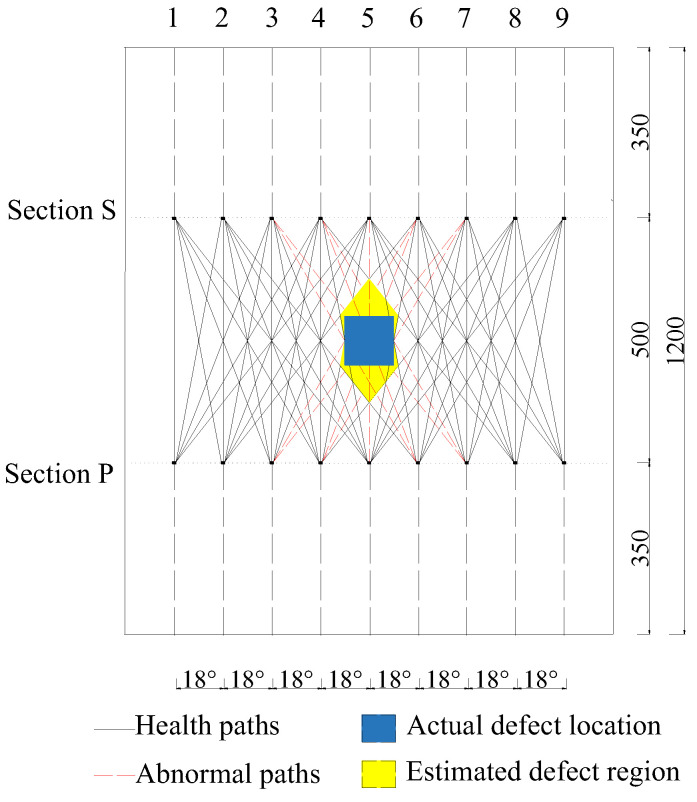
Defect region and abnormal path (unit: mm).

Based on the abnormal surface wave traveling paths determined above, which were identified using surface wave measurements from different traveling paths, the area enclosed by the intersection of these abnormal paths can be judged as the interface debonding area, as shown in Figure 20. It can be observed that the center of the identified debonding defect region aligns closely with the center of the actual defect region, thereby validating the effectiveness of the OPMC configuration of the surface wave method for precise localization. Although a significant discrepancy is evident between the yellow region (estimated defect region) and the blue region (actual defect region) in Figure 20, this is primarily due to the configuration of the measurement paths. In this study, only the bottom-transmit-top-receive surface wave measurement was employed, which resulted in artifacts at the upper and lower parts of the identified region. To achieve more accurate localization of interfacial debonding defects in practical engineering applications, PZT patches can be additionally placed on the left and right sides of the identified region to perform left-transmit-right-receive surface wave measurements. This approach can effectively reduce the discrepancy between the identified region and the actual defect region.

## 5. Conclusions

In this study, a multi-physics analysis of stress wave propagation of underwater GJCs with mimicked interface debonding defects using surface-mounted PZT actuators and sensors was conducted. The generation and propagation of stress waves in the GJCs under the excitation of the surface-mounted PZT actuators with a high-frequency voltage signal were investigated in detail. The effect of different dimensions of mimicked interface debonding defects on the output voltage signals of PZT sensors for different surface stress wave traveling paths was illustrated. The identification and localization of interface debonding defects in the GJCs were achieved based on an OPMC configuration and abnormal surface stress wave traveling path judgment. The main conclusions from this multi-physics coupling simulation are as follows:

(1) To study the mechanism of the interface debonding defect detection approach for underwater GJCs using surface-mounted PZT patches, the influence of interfacial debonding defects on the stress wave propagation within GJC members was investigated using a three-dimensional multi-physics coupling model. This model included surface-mounted PZT patches and an underwater GJC model with mimicked interface debonding defects of different dimensions. Numerical simulations explored the effects of interface debonding defects with different dimensions on the stress wave fields and the corresponding energy distribution within the GJC. Results show that interface debonding alters surface stress wave propagation modes, surface wave fields, and energy attenuation characteristics, thereby elucidating the mechanism of interface debonding defects detection in underwater GJCs based on surface wave measurements.
(2) When a mimicked interface debonding defect is located on the surface wave traveling path of an underwater GJC specimen using an OPOC configuration, the amplitude of the PZT sensor response is noticeably greater than that of PZT sensors located on paths without such defects. The existence of interface debonding defects isolates stress wave propagation and energy loss in the grouting material. As the defect length in the direction of wave propagation increases, the signal amplitude from the PZT sensor also increases. Furthermore, variations in the defect width greater than 50 mm in the circumferential direction do not significantly affect the PZT sensor signal. The output signal from the PZT sensor in the presence of interface debonding exhibits considerable changes compared to that of PZT sensors in healthy surface wave traveling paths, thereby indicating the feasibility of detecting interface debonding using surface wave measurements.
(3) To localize the region of interface debonding in underwater GJCs, an OPMC configuration is proposed with the help of an abnormal surface stress wave traveling path judgment. The time-domain responses of surface-mounted PZT sensors of different surface stress wave traveling paths were simulated using multi-physics coupling analysis. Abnormal values of the WPEs of PZT sensors in each group with identical traveling paths are identified. Based on the effect of interface debonding on surface wave propagation illustrated above, the abnormal surface stress wave traveling paths are identified, and the regions of interface debonding are visualized. The results show that the identified interface debonding regions match well with the location of the mimicked interface debonding defects in the numerical models of the GJCs.

Although the localization method combining surface wave measurement with abnormal value analysis can effectively identify interface debonding defects in underwater GJCs, further investigations are necessary. For instance, the effect of the size of the water area is not considered in the current finite element analysis, and the shape of the interface debonding defects in the model is relatively simple. Therefore, additional research is required to validate the proposed method in the future.

## Figures and Tables

**Table 1:** Material properties.

Material	Density (kg/m³)	Poisson Ratio	Young’s Modulus (GPa)
Steel	7850	0.3	200
Grouting material	2300	0.2	23
Water area	1	—	—

**Table 2:** Interface debonding defect (unit: mm).

	Mimicked Defects Label				
**Defect dimension** **(Height × Circumferential)**	D1	D2	D3	D4	D5
	50 × 100	100 × 100	150 × 100	100 × 50	100 × 150

## Data Availability

Data are contained within the article.
